# Accelerated Recovery of Mitochondrial Membrane Potential by GSK-3β Inactivation Affords Cardiomyocytes Protection from Oxidant-Induced Necrosis

**DOI:** 10.1371/journal.pone.0112529

**Published:** 2014-11-12

**Authors:** Daisuke Sunaga, Masaya Tanno, Atsushi Kuno, Satoko Ishikawa, Makoto Ogasawara, Toshiyuki Yano, Takayuki Miki, Tetsuji Miura

**Affiliations:** Department of Cardiovascular, Renal and Metabolic Medicine, Sapporo Medical University School of Medicine, Sapporo, Japan; Northwestern University, United States of America

## Abstract

Loss of mitochondrial membrane potential (ΔΨ_m_) is known to be closely linked to cell death by various insults. However, whether acceleration of the ΔΨ_m_ recovery process prevents cell necrosis remains unclear. Here we examined the hypothesis that facilitated recovery of ΔΨ_m_ contributes to cytoprotection afforded by activation of the mitochondrial ATP-sensitive K^+^ (mK_ATP_) channel or inactivation of glycogen synthase kinase-3β (GSK-3β). ΔΨ_m_ of H9c2 cells was determined by tetramethylrhodamine ethyl ester (TMRE) before or after 1-h exposure to antimycin A (AA), an inducer of reactive oxygen species (ROS) production at complex III. Opening of the mitochondrial permeability transition pore (mPTP) was determined by mitochondrial loading of calcein. AA reduced ΔΨ_m_ to 15±1% of the baseline and induced calcein leak from mitochondria. ΔΨ_m_ was recovered to 51±3% of the baseline and calcein-loadable mitochondria was 6±1% of the control at 1 h after washout of AA. mK_ATP_ channel openers improved the ΔΨ_m_ recovery and mitochondrial calcein to 73±2% and 30±7%, respectively, without change in ΔΨ_m_ during AA treatment. Activation of the mK_ATP_ channel induced inhibitory phosphorylation of GSK-3β and suppressed ROS production, LDH release and apoptosis after AA washout. Knockdown of GSK-3β and pharmacological inhibition of GSK-3β mimicked the effects of mK_ATP_ channel activation. ROS scavengers administered at the time of AA removal also improved recovery of ΔΨ_m_. These results indicate that inactivation of GSK-3β directly or indirectly by mK_ATP_ channel activation facilitates recovery of ΔΨ_m_ by suppressing ROS production and mPTP opening, leading to cytoprotection from oxidant stress-induced cell death.

## Introduction

Mitochondrial membrane potential (ΔΨ_m_) is crucial for cell viability. Loss of ΔΨ_m_ by opening of the mitochondrial permeability transition pore (mPTP) is a major mechanism of myocardial infarction [1], [2] and cerebral infarction [3] after ischemia/reperfusion. Dissipation of ΔΨ_m_ has also been shown to precede shrinkage and fragmentation of cells, contributing to programed cell death [4], [5]. Loss of ΔΨ_m_ by irreversible opening of the mPTP leads to arrest of mitochondrial ATP synthesis, mitochondrial swelling and outer membrane permeabilization. During ischemia, ΔΨ_m_ is temporarily maintained by consumption of ATP by mitochondrial ATPase, and its recovery after reperfusion depends on ischemia-induced injury of the mitochondrial machinery and on the level of stimuli for mPTP opening upon reperfusion [6], [7]. Involvement of mPTP opening and ΔΨ_m_ loss in reperfusion-induced cell death has been supported by results of studies showing that protection against reperfusion injury afforded by ischemic preconditioning (IPC) and IPC mimetics was associated with inhibition of mPTP opening [1], [8]–[11]. On the other hand, few studies have examined if manipulation of ΔΨ_m_ recovery protects the heart.

A rationale for the hypothesis that acceleration of ΔΨ_m_ recovery protects cells from necrosis has been provided by several lines of evidence. First, preserved ΔΨ_m_ is necessary for mitochondrial ATP synthesis, and recovery of ATP synthesis is crucial for restoration of intracellular Na^+^ and Ca^2+^ homeostasis [5], [7]. Second, there are differences between mitochondria within a cell in susceptibility to Ca^2+^-induced mPTP opening, production of reactive oxygen species (ROS) and structural changes after ischemia/reperfusion [9], [10]. ROS-induced ROS release and Ca^2+^-induced Ca^2+^ release from mitochondria have been reported as mechanisms of accelerated ROS production and Ca^2+^ overload [12]–[14]. Hence, the recovery of ΔΨ_m_ after withdrawal of insults is also likely to be heterogeneous in mitochondria within a cell. Third, mPTP opening is not always irreversible and re-closure of mPTPs after reperfusion has been shown in rat hearts by use of D-^3^H-2-deoxyglucose as a tracer of opened mPTPs [15], [16], suggesting that recovery of ΔΨ_m_ can be achieved by re-closure of mPTPs. Collectively, these findings indicate the possibility that the percentage of mitochondria with unrecoverable ΔΨ_m_ within a cell upon removal of an insult (for example, ischemia/reperfusion) determines mortality of the cell.

We hypothesized that acceleration of ΔΨ_m_ recovery by pro-survival signaling protects myocytes from necrosis. To test this hypothesis, we examined the time course of ΔΨ_m_ recovery in response to a period of exposure to ROS in H9c2 and C2C12 cells and possible modification of the time course by activation of the mitochondrial ATP-sensitive K^+^ (mK_ATP_) channel and by inhibition of glycogen synthase kinase-3β (GSK-3β). The mK_ATP_ channel and GSK-3β were selected for testing effects on ΔΨ_m_ as these two localize within mitochondria and are known to play roles in regulation of the threshold for mPTP opening [2], [17], [18].

## Materials and Methods

### Cell culture and experimental protocols

H9c2 cells, C2C12 cells and human embryonic kidney cells (HEK-293 cells) were obtained from ATCC (American Type Culture Collection). The cells were cultured in DMEM (4.5 g/L glucose) supplemented with 10% fetal bovine serum and antibiotics. All experiments using H9c2 and C2C12 cells were started after serum deprivation for 24 h. An inhibitor of complex III, antimycin A (AA, 40 µM), was used to induce mitochondrial ROS generation. After 60-min treatment with AA, AA was washed out from the culture medium by replacing the medium with pre-warmed fresh medium without AA. Hypoxia/reoxygenation was not employed to induce oxidant stress in this study, since hypoxia alone reduces ΔΨ_m_ and activates multiple pathways of intracellular signaling, which complicates analysis of mPTP-relevant signaling.

To examine the effects of activation of the mK_ATP_ channel, an NO donor and inhibition of GSK-3β, H9c2 cells were incubated with a vehicle, nicorandil (300 µM), diazoxide (300 µM), S-nitroso-N-acetyl-DL-penicillamine (SNAP, 1 µM), or LiCl (30 mM), for 1 h before addition of AA to the medium, and the treatment was continued until the end of the experiment. Treatment with 5-hydroxydecanote (5-HD, 100 µM) to inhibit opening of the mK_ATP_ channel and treatment with mercaptopropionyl glycine (MPG, 30 µM) to suppress ROS during nicorandil treatment were commenced 30 min before treatment with a K_ATP_ channel opener and discontinued with the onset of AA treatment. To examine the possible effect of ROS on recovery of H9c2 cells, MPG (30 µM) or N-acetyl-cysteine (NAC, 1 mM) was added to the medium during AA treatment or to the medium after AA treatment (i.e., a non-AA-containing medium). The effect of inhibition of glycolysis was examined by use of iodoacetate (IAA, 30 µM).

### Monitoring of mitochondrial membrane potential

Mitochondrial membrane potential was monitored by tetramethylrhodamine ethyl ester (TMRE) fluorescence as previously reported [19], [20]. H9c2 cells or C2C12 cells were loaded with TMRE (100 nM) 1 h before AA treatment for assessing the effects of AA on ΔΨ_m_ or at the time of AA washout for assessing recovery of ΔΨ_m_ from oxidant stress by AA. Level of TMRE fluorescence at each time point was expressed as percentage of values in time controls without AA. Fluorescence was recorded by fluorescence microscopy (Olympus IX-70), and images of TMRE fluorescence taken at a magnification of 400× were quantified by pixel counts after cutting off background fluorescence using a threshold value. Data from three regions of interest (ROI) were averaged for each well in the culture plate, and the numbers of cells within the ROI were made comparable between treatment groups. In cells loaded with TMRE after washout of AA, TMRE fluorescence level was normalized by values of time controls in the same culture plate since we had confirmed that TMRE fluorescence level did not significantly change for 3 h of the control period.

### Monitoring of mitochondrial permeability by calcein

In addition to TMRE, a membrane potential-independent tracer, calcein, was used for detection of opening of the mPTP as previously reported [21], [22]. Briefly, in experiments in which the effect of AA on the mPTP was examined, cells were incubated with 0.25 µM calcein for 15 min, and the medium was changed to calcein-free medium containing 4 mM of cobalt chloride (CoCl_2_) before treatment with AA. In experiments in which mPTP opening status after AA washout was examined, calcein was loaded for 15 min immediately after washout of AA. The medium was then replaced with a calcein-free medium containing 4 mM CoCl_2_. Cells were pretreated with 1 µM MitoTracker red for 15 min to stain mitochondria before AA treatment. The calcein-stained area overlapped with the MitoTraker red-stained area was used as an index of mitochondria with closed mPTP and quantified as the level of TMRE described above. Data were normalized by time control cell data. In pilot experiments (n = 6), MitoTracker red was found to be lost from some mitochondria after AA treatment, and 68.3±6.2% of mitochondria retained MitoTracker red at 60 min after washout of AA. Thus, the extent of mPTP opening was somewhat underestimated by the present method.

### Isolation of mitochondria and cytosol fractions, Western blotting, and immunoprecipitation

Mitochondrial and cytosolic fractions of H9c2 cells were prepared by using a mitochondrial isolation kit (Pierce Biotechnology, Rockford, IL) according to the manufacturer's protocol. The samples were subjected to SDS-PAGE, followed by transfer to a polyvinylidene difluoride membrane. After blocking with TBS-T with 5% skim milk or 5% BSA, the membrane was incubated with the primary antibody at 4°C overnight. After incubation with the secondary antibody, the bands were visualized by a standard ECL technique. Interaction of GSK-3β and Reiske was analyzed by immunoprecipitation experiments. Precleared cell lysates (500 µg) were incubated with 2 µg of anti-Rieske antibody in IP buffer (20 mM Tris–HCl [pH 7.4], 1 mM EGTA, 5 mM NaN_3_, 50 mM NaCl, 1 mM PMSF, 50 mM Na_3_VO_4_, 1% Triton X-100, 0.5% NP-40 and a protease inhibitor cocktail) at 4°C overnight with rotation. The antibody-Rieske complex was collected with magnet beads and washed with IP buffer. The immunoprecipitates were subjected to Western blotting as described above. Antibodies used were rabbit monoclonal anti-GSK-3β (#9315, Cell Signaling), rabbit polyclonal anti-phospho-(Ser9) GSK-3β (#9336, Cell Signaling), rabbit polyclonal anti-glycogen synthase (#3893, Cell Signaling), rabbit polyclonal anti-phospho-(Ser641/645) glycogen synthase (44-1092G, Invitrogen), mouse monoclonal anti-Rieske (ab14746, Abcam), goat polyclonal anti-ANT (sc-9299, Santa Cruz), mouse monoclonal anti-VDAC1 (ab14734, Abcam), mouse monoclonal anti-cyclophilin D (AP1035, Calbiochem), mouse monoclonal anti-prohibitin (sc-56346, Santa Cruz), mouse monoclonal anti-β-actin (A5316, SIGMA) and rabbit polyclonal anti-inorganic phosphate carrier (custom-made antibody [23]).

### Transfection of siRNA

Knockdown of GSK-3β was performed by transfection of siRNA against rat GSK-3β (Mission siRNA, SASI_Rn01_00035806, Sigma-Aldrich) using Nucleofection (Lonza Walkersville, MD) according to the manufacturer's protocol. Experiments were completed 48 h after transfection.

### Determination of cell necrosis and apoptosis

Cell necrosis was analyzed by determination of lactate dehydrogenase (LDH) released into the incubation medium. LDH activity in the culture medium and LDH activity in the medium after freeze-thawing of the cells (total cellular LDH activity) were measured by using a CytoTox 96 Non-Radioactive Cytotoxicity assay kit (Promega, Madison, WI) according to the manufacturer's protocol. LDH activity in the medium as a percentage of the total cellular LDH activity was used as an index of cell necrosis. To quantify apoptosis, cells were stained with Hoechst33342 as previously reported [24]. Apoptosis of cells was defined as nuclear condensation revealed by Hoechst33342.

### Determination of ROS production

Intracellular ROS levels were monitored by 2′-7′-dichlorofluorescein (DCF) fluorescence. H9c2 cells were loaded with DCF according to the manufacturer's protocol. DCF fluorescence was recorded by FLoid Cell Imaging Station (Life Technologies, CA) at 30 and 60 min after the start of incubation in an AA-containing medium and at 15 and 60 min after washout of AA.

### Determination of ATP level

H9c2 cells were subjected to 60-min treatment with AA (40 µM), 120-min treatment with IAA (30 µM), combination of AA and IAA treatments or 120-min treatment with a vehicle, and their ATP levels were determined by an assay kit, CellTiter-Glo Luminescent Cell Viability Assay G7570 (Promega, Madison, USA).

### Succinate dehydrogenase activity assay

Cells were subjected to 24-hour serum-deprived culture and then incubated in AA (40 µM)-containing medium or normal medium for 60 min with or without 300 µM nicorandil. For cells treated with both AA and nicorandil, nicorandil was added to the medium 60 min before the onset of incubation with AA. Cellular succinate dehydrogenase (complex II) activity was measured by using a Complex II Enzyme Activity Microplate Assay Kit (Abcam, Cambridge, UK) according to the manufacturer's protocol.

### Chemical compounds

AA, diazoxide, 5-HD, LiCl, MPG, and cyclosporine A were purchased from Sigma Aldrich (St. Louis, MO). NAC was from Wako Pure Chemical Industries (Osaka, Japan). Nicorandil was provided by Chugai Pharmaceutical Co. Ltd. (Tokyo, Japan). TMRE, calcein and DCF were purchased from Invitrogen (Carlsbad, CA).

### Statistical analysis

Data are presented as means ± standard error of the mean. One-way or two-way analysis of variance (ANOVA) was used to detect significant differences between group means in the treatment groups. When ANOVA indicated a significant overall difference, multiple comparisons of the groups were performed by the Student-Newman-Keuls *post-hoc* test. A difference was considered to be statistically significant if p was less than 0.05.

## Results

### Activation of the mK_ATP_ channel promotes recovery of ΔΨ_m_ dissipated by AA

**Figure 1:**
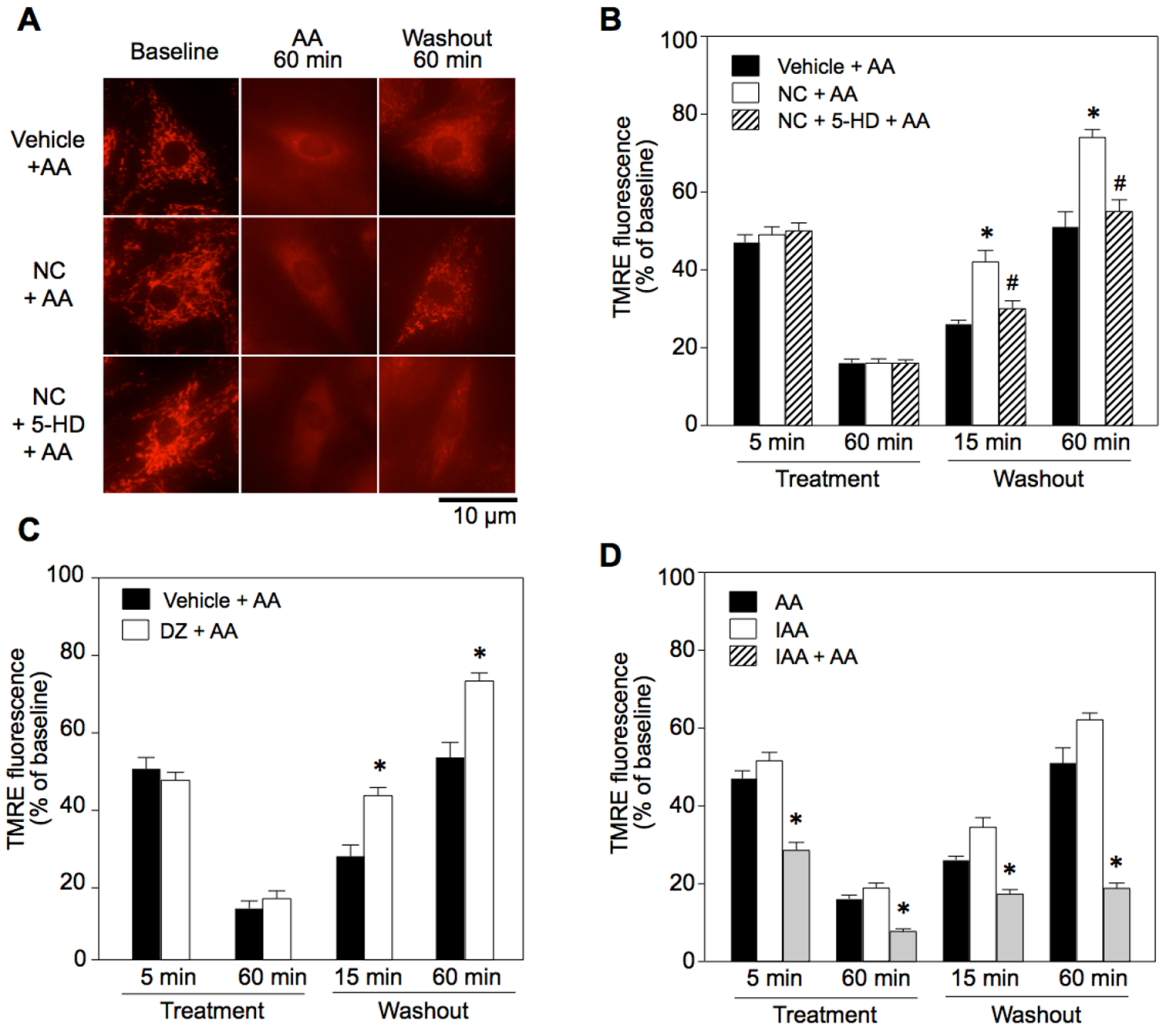
Effects of mK_ATP_ channel openers and a glycolysis inhibitor on antimycin A-induced loss of ΔΨ_m_ and its recovery in H9c2 cells. A: TMRE images before and after antimycin A (AA) treatment (Images were from different cells). B and C: TMRE fluorescence in NC- (B) and DZ-pretreated cells (C). D: TMRE fluorescence in IAA-treated cells. TMRE fluorescence in NC =  nicorandil, DZ =  diazoxide, 5-HD =  5-hydoxydecanote, IAA  =  iodoacetate, Treatment  =  time after onset of treatment with AA, Washout  =  time after washout of AA. *p<0.05 vs. Vehicle+AA or AA, #p<0.05 vs. NC+AA. N = 8.

During treatment with AA, TMRE fluorescence was reduced to 15% of the control and the rod-shaped structure of mitochondria became blurred, indicating reduction in ΔΨ_m_ and mPTP opening (Figure 1). At 60 min after washout of AA, the level of TMRE fluorescence was 51% of the baseline (Figure 1B and C). Since AA not only induces ROS generation but also inhibits oxidative phosphorylation, both effects were possibly responsible for the change in ΔΨ_m._ To assess the impact of ATP deletion on the time course of ΔΨ_m_ after AA treatment, we compared its effect with those of IAA and AA+IAA. AA reduced ATP level to 23.9±1.2% of vehicle controls, and a significantly greater reduction in ATP level was achieved by IAA (13.0±0.4%). The combination of AA and IAA almost completely depleted ATP (0.6±0.2%). Although depletion of ATP was significantly less in the AA-treated group, time courses of TMRE fluorescence were similar in the AA-treated and IAA-treated groups (Figure 1D). Together with the effects of ROS scavengers on TMRE fluorescence (see “Relationship between ROS and recovery of ΔΨ_m_” below), these results indicate that ROS, in addition to ATP depletion, was responsible for loss of ΔΨ_m._ by AA treatment.

**Figure 2:**
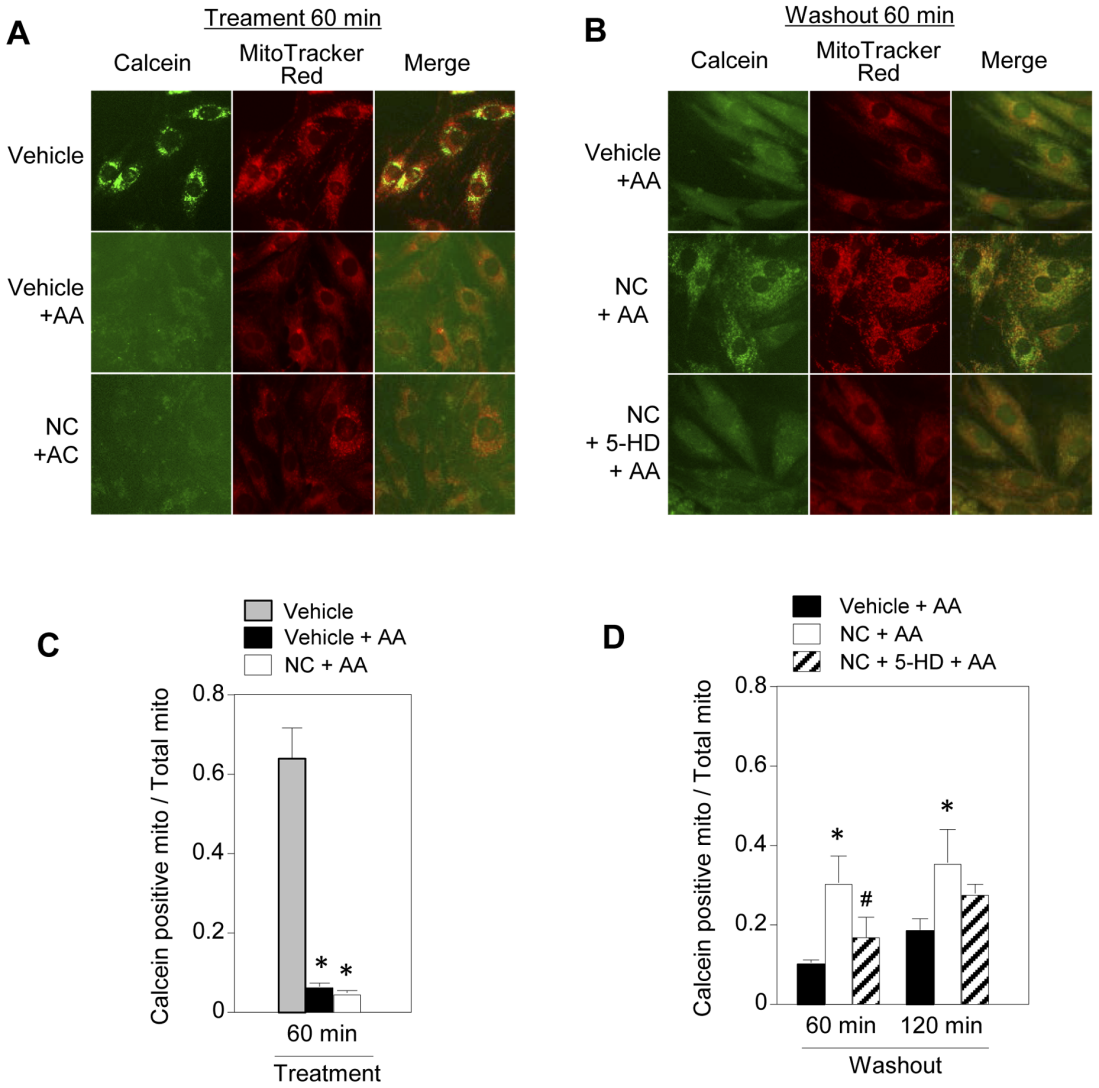
Effects of mK_ATP_ channel openers on antimycin A-induced mPTP opening and its recovery in H9c2 cells. A and B: Calcein and MitoTracker images before (A) and after (B) antimycin A (AA) treatment. C and D: Levels of calcein-positive mitochondria 60 min after AA treatment (C) and 60 and 120 min after washout of AA (D). Level of calcein-positive mitochondria is expressed as the ratio of calcein-positive area to MitoTracker-positive area. *p<0.05 vs. Vehicle+AA, #p<0.05 vs. NC+AA.

Opening of the mPTP by AA was indicated by the finding that calcein loaded in mitochondria leaked into the cytosol after AA treatment (Figure 2A). The ratio of mitochondria positive for calcein was 6% of the baseline at 60 min after AA treatment, and the ratios were 10% and 18% of time control value at 60 and 120 min after AA washout, respectively (Figure 2B and C). In the vehicle-treated controls, the ratio of mitochondria positive for calcein was not 100% (Figure 2C) since the threshold for calcein fluorescence was set at a relatively high level in order to include clearly discrete mitochondrial calcein.

**Figure 3:**
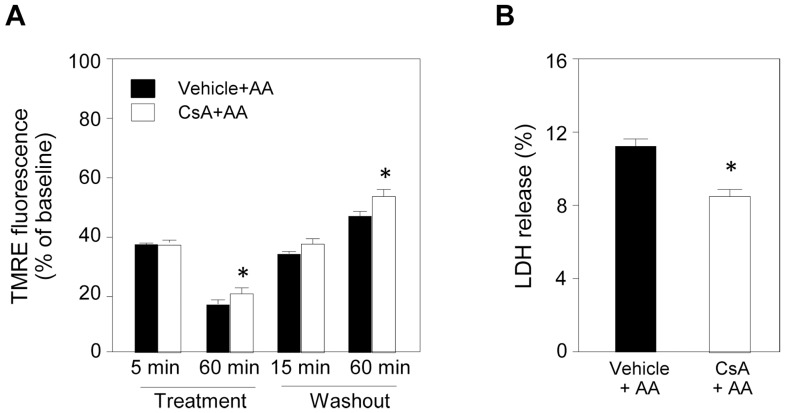
Effects of cyclosporine A on antimycin A-induced changes in ΔΨ_m_ and LDH release. A: Level of TMRE fluorescence was determined as an index of ΔΨ_m_. Cyclosporine A (CsA, 0.5 µM) was added to the medium 60 min before antimycin A (AA) treatment. AA-induced reduction of TMRE fluorescence at 60 min after the onset of AA treatment was slightly attenuated in the CsA-treated group compared to that in the AA group, indicating partial suppression of ROS-induced mPTP opening. Recovery of TMRE fluorescence at 60 min after washout of AA was also slightly improved by CsA. N = 5 per group. B: LDH released after washout of AA was significantly reduced by CsA. *p<0.05 vs. Vehicle+AA. N = 8 per group.

Contribution of mPTP opening to reduction in TMRE fluorescence and cell necrosis after washout of AA was supported by results showing that cyclosporine A, a direct mPTP inhibitor, attenuated the effect of AA treatment on TMRE fluorescence and LDH release after AA washout by 24% (Figure 3). Protein levels of putative regulatory subunits of the mPTP (adenine nucleotide translocase, voltage-dependent anion channel, inorganic phosphate carrier and cyclophilin D) were not changed by AA (Figure S1).

**Figure 4:**
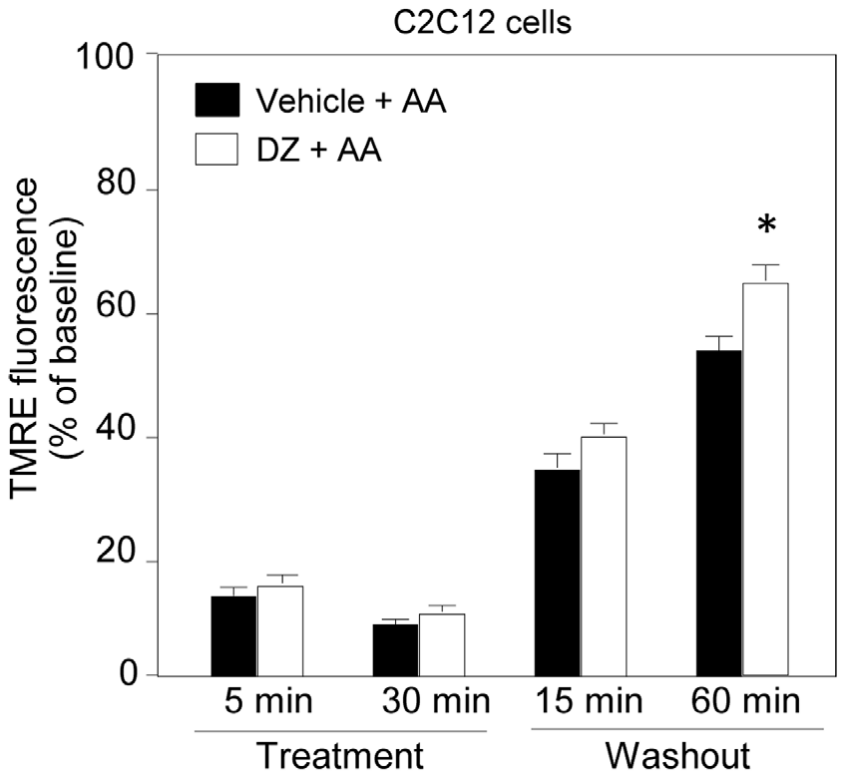
Effects of an mK_ATP_ channel opener on antimycin A-induced loss of ΔΨ_m_ and its recovery in C2C12 cells. TMRE fluorescence in untreated and diazoxide-pretreated cells. AA =  antimycin A, DZ =  diazoxide, Treatment  =  time after onset of treatment with AA, Washout  =  time after washout of AA. *p<0.05 vs. Vehicle+AA. N = 8.

Pretreatment with an mK_ATP_ channel activator (nicorandil or diazoxide) did not affect reduction of TMRE fluorescence during AA treatment but significantly enhanced its recovery after washout of AA (Figure 1B and C). This effect of mK_ATP_ channel openers was abolished by 5-HD, an mK_ATP_ channel blocker (Figure 1B). SNAP, an NO donor, did not mimic the effect of nicorandil on TMRE fluorescence after AA treatment, indicating that the nitrate property of nicorandil was not involved in its effect on ΔΨ_m_ (Figure S2). Although SNAP at relatively high doses (0.1–1 mM) has been shown to activate the mK_ATP_ channel [25], [26], we selected a low dose (1 µM) to avoid the effect on the mK_ATP_ channel in this study. Improvement by diazoxide in recovery of TMRE fluorescence after washout of AA was also confirmed in C2C12 cells (Figure 4), indicating that role of the mK_ATP_ channel in ΔΨ_m_ regulation is not unique to H9c2 cells.

### Inhibition of GSK-3β by activation of the mK_ATP_ channel

**Figure 5:**
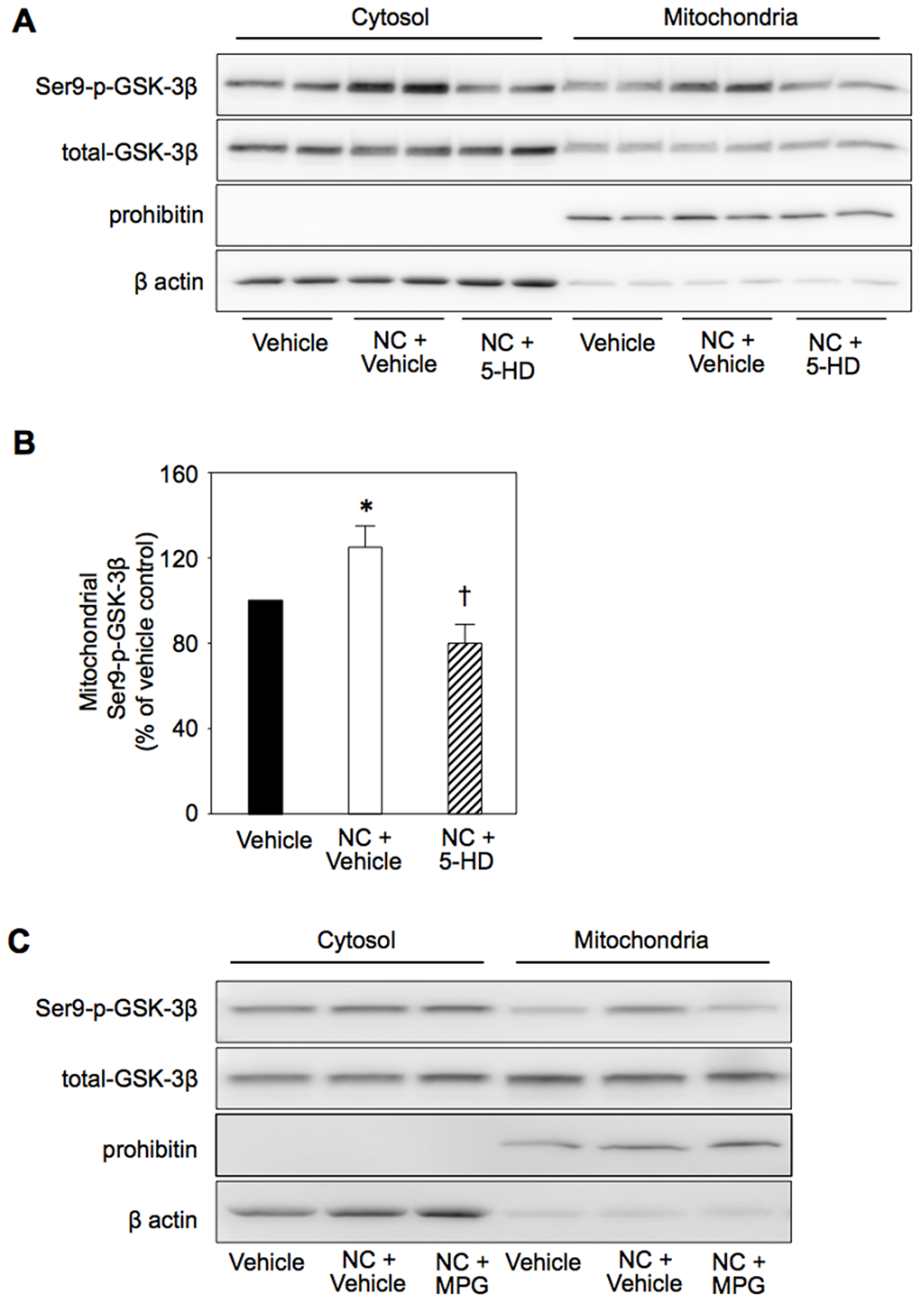
Effects of nicorandil and LiCl on GSK-3β phosphorylation. A: Representative Western blotting for Ser9-phospho-GSK-3β and total GSK-3β in cytosolic and mitochondrial fractions of vehicle-treated, nicorandil-treated (NC) and 5-hydroxydecanoate (5-HD) plus NC-treated cells. B: Group means of mitochondrial Ser9-phospho-GSK-3β levels. NC and 5-HD were added to the culture medium 60 min and 90 min before collection of cells for Western boltting, respectively. *p<0.05 vs. Vehicle, †p<0.05 vs. NC+Vehicle. N = 8 per group. C: Effects of MPG (mercaptopropionyl glycine) on the effect of NC-induced phosphorylation of GSK-3β.

**Figure 6:**
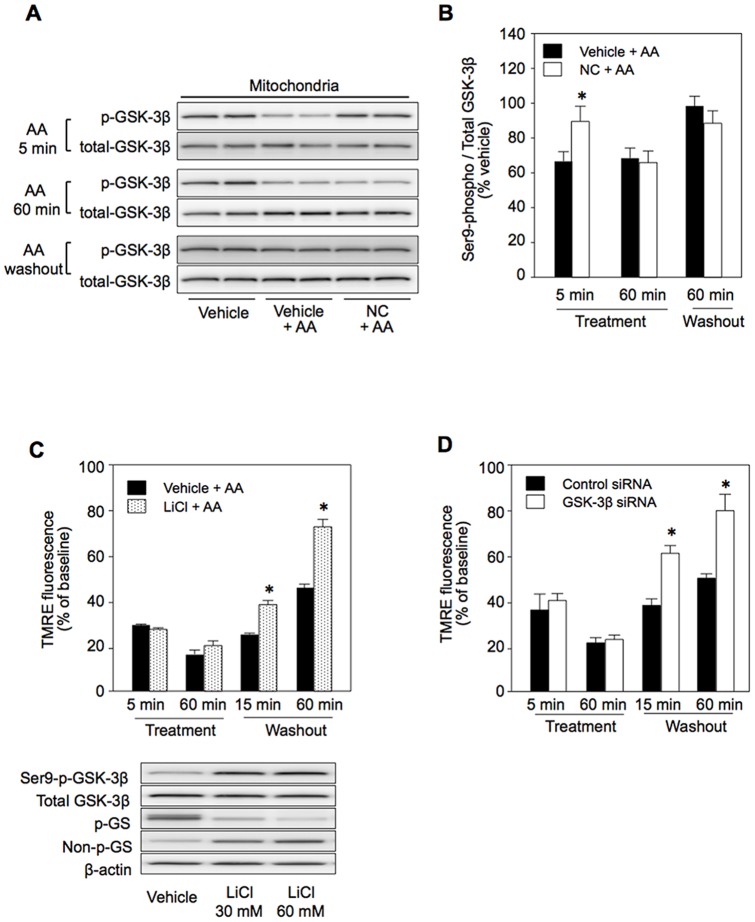
Effects of inhibition of GSK-3β on antimycin A-induced changes in ΔΨ_m_ and its recovery. A: Western blotting for Ser9-phospho- and total GSK-3β in mitochondria, B: Effects of antimycin A (AA) on phospho-GSK-3β level. C: TMRE fluorescence after AA treatment in vehicle- and LiCl-pretreated cells. Western blotting for Ser9-phospho-GSK-3β, total GSK-3β, Ser641/645-phospho-glycogen synthase (GS), non-phospho-GS and β-actin (loading control) in total lysates of vehicle-treated and LiCl-treated cells. Treatments with 30 mM and 60 mM LiCl for 60 min induced phosphorylation of GSK-3β and dephosphorylation of GS. Increased phosphorylation of GSK-3β by LiCl reflects reduced activity of protein phosphatase 1, which is positively regulated by GSK-3β activity. D: TMRE fluorescence after AA treatment in control siRNA- and GSK-3β-siRNA-pretreated cells. NC =  nicorandil. Treatment  =  time after onset of treatment with AA, Washout  =  time after washout of AA. *p<0.05 vs. Vehicle or Control siRNA. N = 8.

Like diazoxide in our previous study [27], nicorandil increased the levels of Ser9-phospho-GSK-3β in the mitochondria and cytosol by 24% and 37%, respectively (Figure 5AB). Phosphorylation of GSK-3β in mitochondria by nicorandil was inhibited by MPG (Figure 5C), indicating that ROS generated by mK_ATP_ channel activation [28], [29] mediated GSK-3β phosphorylation. Elevation of mitochondrial phospho-GSK-3β level by nicorandil was maintained at 5 min after AA treatment, though the effect was not significant afterwards (Figure 6A and B). If inactivation of GSK-3β by phosphorylation at Ser9 contributes to facilitated recovery of ΔΨ_m_ after AA treatment by nicorandil, the effect of nicorandil should be mimicked by an inhibitor of GSK-3β, LiCl, and by knockdown of GSK-3β. That was indeed the case as shown in Figure 6C and D. Inhibition of GSK-3β activity by LiCl was confirmed by the results that LiCl increased Ser9-phospho-GSK-3β level, reflecting suppression of a GSK-3β activity-dependent phosphatase, and reduced phosphorylation of glycogen synthase (GS), a downstream target of GSK-3β (Figure 6C, lower panel).

### Relationship between ROS and recovery of ΔΨ_m_

**Figure 7:**
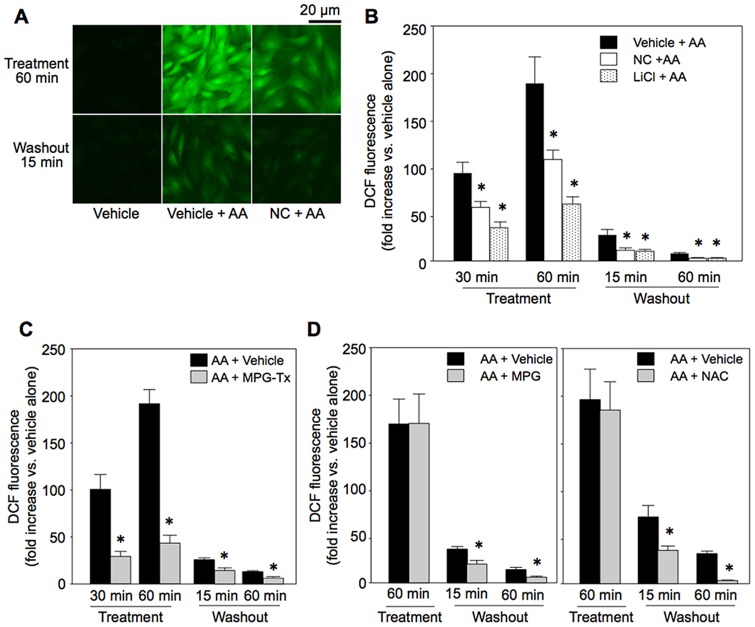
ROS generated by antimycin A. A: Representative DCF images. B: DCF fluorescence during and after antimycin A (AA) treatment in vehicle-, NC- and LiCl-pretreated cells. C: Effects of MPG administered during AA treatment on DCF. D: Effects of MPG or NAC treatment commenced at the time of AA washout on DCF. MPG-Tx  =  treatment with mercaptopropionyl glycine (MPG) during AA treatment, MPG =  MPG treatment commenced at the time of AA washout, NAC =  N-acetylcysteine treatment commenced at the time of AA washout. Treatment  =  time after onset of treatment with AA, Washout  =  time after washout of AA. *p<0.05 vs. Vehicle+AA or AA+Vehicle. N = 8.

The level of ROS determined by DCF was significantly elevated during AA treatment and then decreased time-dependently after washout of AA (Figure 7A and B). Treatment with nicorandil or LiCl reduced the level of ROS during AA treatment and after AA washout (Figure 7B). Although inhibition of the activity of succinate dehydrogenase was reported as a mechanism by which mK_ATP_ channel activation reduces ROS production in the heart [30], succinate dehydrogenase activity in H9c2 cells was not significantly changed by nicorandil or AA: 0.53±0.11 (mOD/min) in controls (vehicle-treated cells), 0.61±0.07 in AA-treated cells, 0.67±0.20 in nicorandil-treated cells and 0.69±0.08 in nicorandil plus AA-treated cells (n = 5 in each treatment).

**Figure 8:**
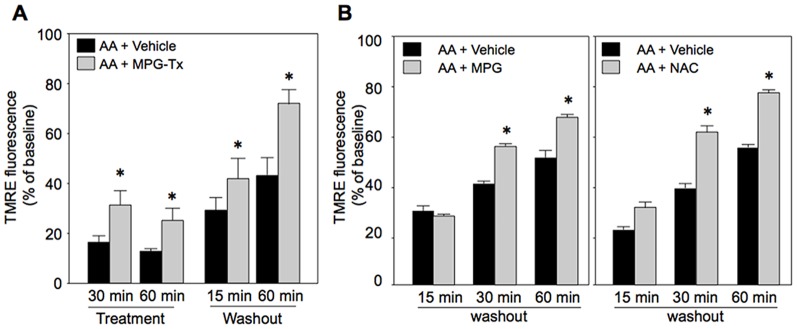
Effects of ROS scavengers on time course of ΔΨ_m_. A and B: Effects of MPG administered during AA treatment (A) and effects of treatment with MPG or NAC commenced at the time of AA washout (B) on TMRE fluoresence. MPG-Tx  =  treatment with mercaptopropionyl glycine (MPG) during AA treatment, MPG  =  MPG treatment commenced at the time of AA washout, NAC  =  N-acetylcysteine treatment commenced at the time of AA washout. Treatment  =  time after onset of treatment with AA, Washout  =  time after washout of AA. *p<0.05 vs. AA+Vehicle. N = 8.

To determine whether ROS during AA treatment or residual ROS being produced after washout of AA inhibit recovery of ΔΨ_m_, we tested the effects of an ROS scavenger during AA treatment or after washout of AA. MPG (30 µM) added to the medium only during AA treatment period significantly reduced ROS both during and after AA treatment (Figure 7C). The effect of MPG on ROS was associated with partial preservation of TMRE fluorescence during AA treatment and improved recovery of TMRE fluorescence (Figure 8A). Treatment with MPG or NAC (1 mM) commenced at the time of washout of AA, which reduced ROS level (Figure 7D), also significantly improved recovery of TMRE fluorescence (Figure 8B), indicating contribution of persistent ROS production after washout of AA to continual mPTP opening.

### Effect of facilitated ΔΨ_m_ recovery on cell necrosis and apoptosis

**Figure 9:**
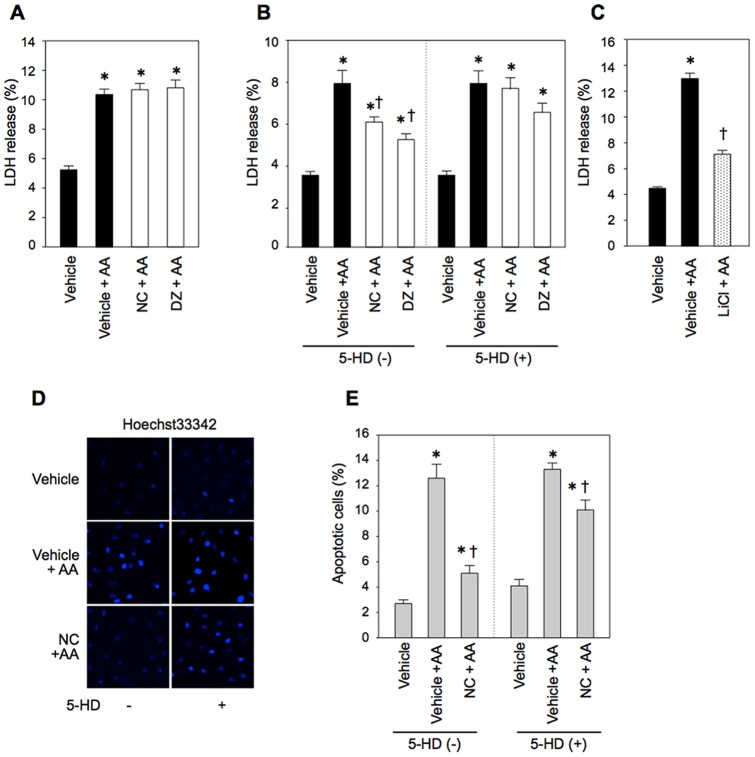
Necrosis and apoptosis after antimycin A treatment. A–C: Cell necrosis indicated by LDH release. LDH release at the end of AA treatment (A) and during a 2-h period after washout of AA (B, C) are shown. D and E: representative images of nuclear staining with Hoechst33342 (D) and apoptosis at 2 h after washout of AA (E). AA =  antimycin A, NC =  nicorandil, DZ =  diazoxide, 5-HD  =  5-hydroxydecanoate. *p<0.05 vs. Vehicle, †p<0.05 vs. Vehicle+AA. N = 8∼12.

LDH released into the medium was determined at the end of AA treatment and at 2 h after AA removal with or without mK_ATP_ channel opener pretreatment. As shown in Figure 9A, LDH release at the end of AA treatment was not reduced by nicorandil or diazoxide. However, LDH release after removal of AA was significantly reduced by nicorandil, diazoxide and LiCl (Figure 9B and C), and the protective effects of mK_ATP_ channel openers were inhibited by 5-HD. Apoptosis after washout of AA was also suppressed by nicorandil in a 5-HD-sensitive manner (Figure 9D and E). Collectively, these results indicate that accelerated recovery of ΔΨ_m_ after oxidant stress by suppression of ROS production via GSK-3β inactivation prevents cell necrosis and apoptosis.

### Modification of mitochondrial complex III by activation of the mK_ATP_ channel

**Figure 10:**
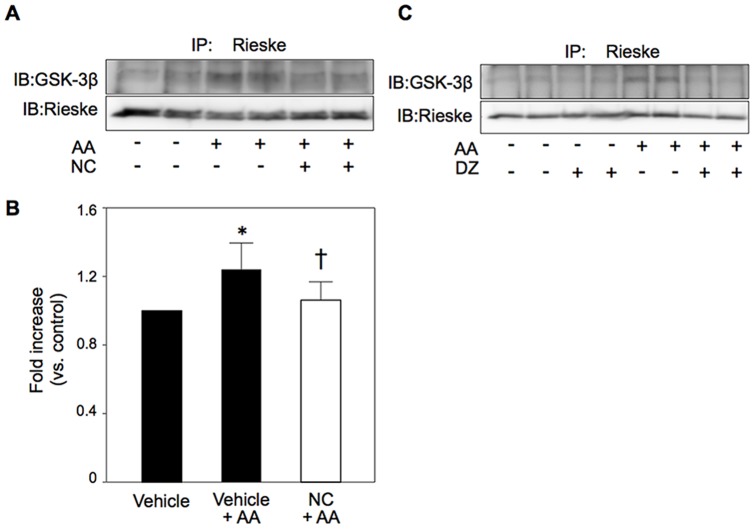
Interaction of Rieske protein and GSK-3β. Cell lysates after 5 min of AA treatment were immunoprecipitated with anti-Rieske antibodies and used for Western blotting for GSK-3β. A and B: Interaction of Rieske with GSK-3β was increased by antimycin A (AA), and the AA-induced changes were attenuated by nicorandil (NC) in H9c2 cells. N = 10∼11 per group. *p<0.05 vs. Vehicle, †p<0.05 vs. Vehicle+AA. C: Similar results were obtained in HEK293 cells by use of diazoxide (DZ).

Since AA-induced ROS production was suppressed by LiCl or nicorandil (Figure 7B), we examined the interaction of complex III, a target for AA to generate ROS, and GSK-3β. Interaction of a complex III subunit, Rieske protein (Rieske), and GSK-3β was significantly increased by AA. The Rieske-GSK-3β interaction was attenuated by mK_ATP_ channel openers in H9c2 cells and also in HEK293 cells (Figure 10).

## Discussion

The time course of ΔΨ_m_ recovery or mPTP re-closure and their relationships with development of tissue injury have not been characterized. The present study showed that activation of the mK_ATP_ channel (Figures 1BC and 4), reduction of GSK-3β activity (Figure 6C and D) or suppression of persistent ROS production (Figure 8B) significantly improved recovery of ΔΨ_m_ from ROS-induced dissipation. Furthermore, the improved ΔΨ_m_ recovery was associated with suppressed LDH release during the recovery process and apoptosis (Figure 9). These results support the notion that facilitation of ΔΨ_m_ recovery protects cell from cell death.

ROS production induced by AA, an inhibitor of complex III, was suppressed by inactivation of GSK-3β (Figure 6), indicating that this kinase enhanced ROS prodcution. Involvement of GSK-3β in mitochondrial ROS production is not specific to AA-induced ROS production. Our recent study has shown that mitochondrial translocation of GSK-3β triggered by exogenous hydrogen peroxide induced enhanced ROS production and that both mitochonrial translocation of GSK-3β and ROS production were dependent on GSK-3β kinase activity [20].

Since opening of the mPTP is a major mechanism of failure of ΔΨ_m_ recovery after ischemia/reperfusion [2], [3], [5], [10], we assessed the impact of mK_ATP_ channel activation on mPTP opening and re-closure. As expected from massive ROS production by AA, the level of mPTP opening was unaffected by activation of the mK_ATP_ channel. While level of ROS production during AA treatment was different between vehicle-treated and nicorandil-treated cells (Figure 7B), TMRE fluorescence was similarly suppressed by AA to 20% of baseline level in both treatment groups (Figure 1B), suggesting presence of ROS threshold level for collapsing ΔΨ_m_. However, level of the calcein-loadable mitochondria was higher in the nicorandil-pretreated group at 60 and 120 min after washout of AA (Figure 2D). Since MitoTracker red is a ΔΨ_m_-dependent probe, use of this probe for identifying calcein localized in mitochondria probably underestimated mitochondria with closed mPTPs after washout of AA. In fact, MitoTracker positive area was reduced to 70∼80% of time control after AA treatment. Nevertheless, there was a significant difference between levels of calcein-positive mitochondria in nicorandil-treated and untreated cells. These findings are consistent with the notion that facilitated re-closure of mPTPs by mK_ATP_ channel activation contributed to improved recovery of ΔΨ_m_ from ROS-induced collapse. However, the possibility that improved preservation of respiratory chain complexes was involved in better recovery of ΔΨ_m_, leading to restoration of mPTP status, cannot be excluded.

As a possible mechanism by which nicorandil promoted re-closure of the mPTP, we postulated that withdrawal of mPTP-opening stimuli after washout of AA was facilitated by nicorandil. Of the known mPTP opening factors [2], we focused on ROS and found that nicorandil and a GSK-3β inhibitor, LiCl, suppressed ROS production during AA treatment and after washout of AA. The effects of these agents on ROS level are consistent with results of previous studies showing that mK_ATP_ channel openers suppress burst production of ROS upon reperfusion in isolated perfused hearts [30], [31] and that a mitochondria-targeting GSK-3β mutant increased ROS production in SH-SY5Y cells [32]. Interestingly, treatment with ROS scavengers only after removal of AA was sufficient to reproduce the effects of pretreatment with nicorandil or LiCl on ΔΨ_m_ (Figure 8B). In contrast, suppression of ROS during AA treatment resulted in parallel up-ward shift of TMRE fluorescence throughout experiments (Figure 8A). These results indicate that suppression of ROS production by inactivation of GSK-3β mediates facilitation of ΔΨ_m_ recovery, possibly via mPTP re-closure, by mK_ATP_ channel activation.

Nicorandil induced phosphorylation of GSK-3β at Ser9 in H9c2 cells (Figures 5 and 6) as did diazoxide in the rat myocardium *in vivo* [27], though its level declined during AA treatment. Interestingly, despite its transient effect on GSK-3β during the early phase of AA treatment, nicorandil improved recovery of ΔΨ_m_ similarly to inactivation of GSK-3β by LiCl throughout the AA treatment period (Figure 1). Hence, a signal mechanism downstrem of this kinase needs to be postulated for suppression of ROS production. However, relationships between mitochondrial GSK-3β and molecules regulating ROS production or ROS elimination remain unclear. A possible explanation is involvement of GSK-3β in persistent ROS production triggered by inhibitionn of comlex III at the Qi site. Recent studies by Viola et al. [33], [34] indicated that transient exposure of cardiomyocytes to hydrogen peroxide induced persistent ROS production by modification of the Qo site of complex III. In addition, ROS-induced ROS release, which potentially leads to chain reactions of mitochondrial ROS production, has been reported [14]. Hence, it is possible that inactivation of GSK-3β during the early phase of AA treatment has some impact on the level of persistent ROS production afterwards. Although inhibition of succinate dehydrogenase activity has been reported as a mechanism of ROS suppression by mK_ATP_ channel openers in rat mitochondria [30], we could not detect a significant change in succinate dehydrogenase activity by nicorandil in the present H9c2 cell preparation.

The mechanism by which inactivation of GSK-3β suppresses ROS production remains unclear. Interestingly, we found that interaction of GSK-3β with complex III was induced by AA in association with ROS production and that mK_ATP_ channel openers suppressed both GSK-3β-Rieske interaction and ROS production by AA. AA induces ROS production by interaction with the Qi site of the cytochrome bc_1_ complex, and Rieske is involved in ROS production in complex III [35]–[37]. Reiske is a major subunit of complex III, and its deletion does not prevent assembly of other submits but abolishes enzymatic activity [37]. Deletion of Rieske also leads to reduction in protein levels of complexes I and IV. ROS production in mitochondria are reportedly increased by Rieske knockout fibroblasts [37], but ROS production during hypoxia in pulmonary artery smooth muscle cells has been shown to be reduced by deletion of Rieske [36]. Modification of the expression levels of complexes I and IV may be involved in the apparently opposite effects of deletion of Rieske on ROS production.

GSK-3β translocates from the cytosol to mitochondria and interacts with adenine nucleotide translocase, a protein in the mitochondrial inner membrane, after ischemia/reperfusion [20], [38]. A role of GSK-3β translocated to mitochondria in ROS production is supported by the finding that selective expression of unregulated GSK-3β in mitochondria significantly increased ROS production in SH-SY5Y cells [32]. However, whether GSK-3β-Rieske interaction is indeed causally related to ROS production at complex III remains to be tested in future projects.

There are limitations in the present study. First, cell necrosis induced by the present dose of AA was modest, and the impact of improvement of ΔΨ_m_ recovery on cell survival has not been characterized fully. However, this is a technical limitation and larger doses of AA used in preliminary experiments increased massive cell necrosis during AA treatment, making it difficult to analyze recovery of ΔΨ_m_ in cells that survived after washout of AA. Second, levels of mPTP opening after washout of AA (Figure 2D) determined by the present method are presumably underestimated since MitoTracker red, a marker of mitochondria, was lost from some mitochondria during AA treatment. However, a significant increase in the percentage of calcein-positive mitochondria after washout of AA was shown, and the results still support the notion that mPTPs re-close after withdrawal of oxidant stress. Third, we mainly used H9c2 cells, a rat cardiomyoblast cell line, and it is unclear whether the present results can be extrapolated to adult cardiomyocytes. However, the effects of mK_ATP_ channel activation on ΔΨ_m_ recovery and on Rieske-GSK-3β interaction were also observed in C2C12 cells and HEK293 cells, respectively, excluding the possibility that response of ΔΨ_m_ to ROS and its modification by activation of the mK_ATP_ channel is unique to H9c2 cells.

In conclusion, the results indicate that facilitated recovery of ΔΨ_m_ from ROS-induced injury can be achieved by inactivating GSK-3β directly or indirectly by activation of the mK_ATP_ channel in isolated cardiomyocytes. The improvement of ΔΨ_m_ recovery protects cardiomyocytes from necrosis by burst production of ROS. Significance of this mechanism in the myocardium *in vivo* remains to be investigated.

## Supporting Information

Figure S1 **Effects of antimycin A on protein levels of putative subunits of the mPTP.**
(DOC)

Figure S2 **Effects of S-nitroso-N-acetyl-DL-penicillamine on antimycin A-induced changes in ΔΨm.**
(DOC)
