# Supplementary material for: Accelerated Recovery of Mitochondrial Membrane Potential by GSK-3β Inactivation Affords Cardiomyocytes Protection from Oxidant-Induced Necrosis
Source: PLoS One. 2014 Nov 12;9(11):e112529. doi: 10.1371/journal.pone.0112529 (PMC4229200; doi:10.1371/journal.pone.0112529)
Supplement: Figure S1 — Effects of antimycin A on protein levels of putative subunits of the mPTP. (DOC) [file pone.0112529.s001.doc]

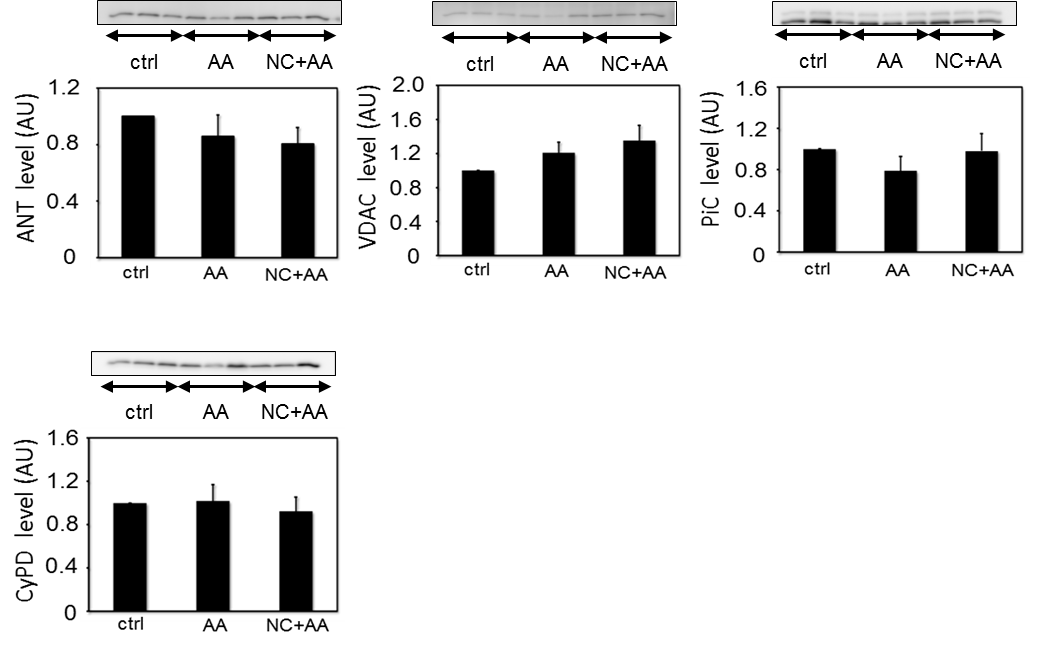


Figure S1. Effects of antimycin A (AA) on protein levels of putative subunits of the mPTP.

Protein levels of adenine nucleotide translocase (ANT), voltage-dependent anion channel (VDAC), inorganic phosphate carrier (PiC) and cyclophilin D (CypD) in H9c2 cells were normalized by loading controls and then expressed as ratios to means in the vehicle controls. There was no significant difference in ANT, VADC, PiC or CypD between three treatment groups. ctrl = 120 min treatment with vehicle, Vehicle+AA = treatment with antimycin A (AA, 40 μM) for 60 min after addition of vehicle to the medium, NC+vehicle = treatment with AA for 60 min after 60-min pretreatment with nicorandil (300 μM). a.u. = arbitrary unit. N = 5~6 per group.
