# Supplementary material for: Accelerated Recovery of Mitochondrial Membrane Potential by GSK-3β Inactivation Affords Cardiomyocytes Protection from Oxidant-Induced Necrosis
Source: PLoS One. 2014 Nov 12;9(11):e112529. doi: 10.1371/journal.pone.0112529 (PMC4229200; doi:10.1371/journal.pone.0112529)
Supplement: Figure S2 — Effects of S-nitroso-N-acetyl-DL-penicillamine on antimycin A-induced changes in ΔΨm. (DOC) [file pone.0112529.s002.doc]

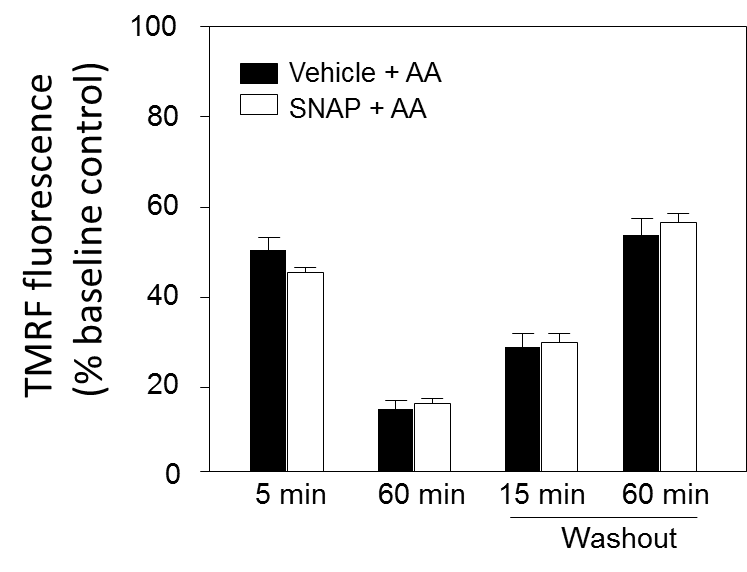


Figure S2. Effects of S-nitroso-N-acetyl-DL-penicillamine (SNAP) on AA-induced changes in ∆Ψ_m_.

As an index of ∆Ψ_m_, TMRE fluorescence was determined at 5 and 60 min after the onset of AA treatment and at 15 min and 60 min after washout of AA. Treatment with a vehicle or SNAP (1 μM) was commenced 60 min before AA-treatment and continued until the end of the experiment. AA = antimycin A, Treatment = time after the onset of treatment with AA, Washout = time after washout of AA. N = 8 per group.
